# Le syndrome d'Aicardi-Goutières avec présentation atypique: mutation du gène RNASEH2B chez un nourrisson sans microcéphalie ni calcifications intracrâniennes (à propos d’un cas)

**DOI:** 10.11604/pamj.2025.51.102.48730

**Published:** 2025-08-21

**Authors:** Samir Iddir, Brahim Lounis, Massissilia Ouarab, Thanina Guendoud, Channez Feghoul, Karima Benarab, Athmane Hamzaoui, Nadia Benssadi

**Affiliations:** 1 Service de Pédiatrie, Centre Hospitalier Universitaire Nadir Mohammed, Tizi-Ouzou, Algérie,; 2 Faculté de Médecine, Université Mouloud Mammeri, Tizi-Ouzou, Algérie

**Keywords:** Aicardi-Goutières syndrome, psychomotor regression, RNASEH2B mutation, genetic encephalopathy, case report, Syndrome d'Aicardi-Goutières, régression psychomotrice, mutation RNASEH2B, encéphalopathie génétique, cas clinique

## Abstract

Le syndrome d'Aicardi-Goutières (SAG) représente une encéphalopathie génétique rare fréquemment confondue avec d'autres pathologies neurologiques pédiatriques. Nous rapportons le cas d'un nourrisson de 14 mois ayant développé une régression psychomotrice à partir de l'âge de 6 mois, avec une présentation atypique caractérisée par l'absence de microcéphalie et de calcifications intracrâniennes. L'imagerie par résonance magnétique (IRM) cérébrale a révélé uniquement une légère atrophie cortico-sous-corticale. Après exclusion systématique des étiologies infectieuses et métaboliques, l'analyse génétique a identifié une mutation homozygote du gène RNASEH2B, confirmant le diagnostic de SAG. Ce cas démontre l'hétérogénéité phénotypique du syndrome et l'importance cruciale du séquençage génétique dans le diagnostic des encéphalopathies précoces d'origine indéterminée, même en l'absence des marqueurs cliniques et radiologiques classiques.

## Abstract (en)

Aicardi-Goutières syndrome (AGS) is a rare genetic encephalopathy frequently misdiagnosed as other pediatric neurological conditions. We report the case of a 14-month-old infant who developed psychomotor regression from the age of 6 months, exhibiting an atypical presentation characterized by the absence of microcephaly and intracranial calcifications. Cerebral MRI revealed only mild cortico-subcortical atrophy. Following systematic exclusion of infectious and metabolic etiologies, genetic analysis identified a homozygous mutation in the RNASEH2B gene, confirming the diagnosis of AGS. This case highlights the phenotypic heterogeneity of the syndrome and the crucial importance of genetic sequencing in diagnosing early-onset encephalopathies of undetermined origin, even in the absence of classic clinical and radiological markers.

## Introduction

Le SAG constitue une encéphalopathie génétique rare, initialement décrite en 1984 par Aicardi *et al*. [1]. Cette affection est caractérisée par une signature interféron de type I, résultant d'une activation anormale de l'immunité innée en réponse à des acides nucléiques endogènes [2]. Son incidence est estimée à moins de 1 cas pour 100 000 naissances, avec une transmission principalement autosomique récessive [3]. Le SAG se présente cliniquement selon deux phénotypes: une forme néonatale sévère évoquant une infection congénitale et une forme tardive avec régression psychomotrice entre 3 et 12 mois après une phase de développement normal [4]. Le tableau typique associe une encéphalopathie précoce, une microcéphalie progressive, des calcifications des noyaux gris centraux, une leucodystrophie, ainsi qu'une lymphocytose chronique du liquide céphalo-rachidien (LCR) avec élévation de l'interféron alpha [1]. Des signes extraneurologiques peuvent inclure des engelures, une hépatosplénomégalie et des manifestations auto-immunes, notamment un lupus érythémateux systémique [5].

Actuellement, sept gènes sont impliqués dans la pathogénie du SAG (TREX1, RNASEH2A, RNASEH2B, RNASEH2C, SAMHD1, ADAR1 et IFIH1 [6,7], codant pour des protéines essentielles au métabolisme des acides nucléiques. Leur dysfonctionnement entraîne une accumulation anormale d'acides nucléiques endogènes, déclenchant ainsi une cascade inflammatoire médiée par l'interféron alpha. Nous rapportons ici un cas atypique de SAG, dépourvu des signes cardinaux habituellement décrits, illustrant les défis diagnostiques associés à cette pathologie.

## Patient et observation

**Présentation du patient:** nous rapportons le cas d'un nourrisson de sexe féminin âgée de 14 mois, première née de parents non consanguins, sans antécédents familiaux de pathologies neurologiques. La grossesse s'est déroulée normalement jusqu'à terme. L'accouchement a été réalisé par césarienne pour circulaire du cordon, avec d'excellents scores d'Apgar (9/10 à 1 minute, 10/10 à 5 minutes). L'anamnèse révèle un retard vaccinal concernant les vaccins prévus aux 11^e^ et 12^e^ mois.

**Résultats cliniques:** l'examen clinique initial a mis en évidence une irritabilité prononcée associée à des troubles de l'alimentation (mastication insuffisante et difficile) et un stridor laryngé. L'évaluation neurologique a confirmé un retard développemental significatif: absence de station debout avec appui, position assise non acquise, absence de ramper et préhension imprécise. Une hypotonie axiale légère était présente, contrastant avec une préservation de la poursuite oculaire et sonore. Fait notable, plusieurs signes cliniques habituellement associés au SAG étaient absents: périmètre crânien normal (47 cm), absence de spasticité et de convulsions, absence d'engelures aux extrémités et d'hépatosplénomégalie. Le contact social demeurait relativement préservé sans syndrome de dysmorphie apparent. Sur le plan staturo-pondéral, l'enfant présentait une taille normale (76 cm) mais un poids limite (8300 g, entre le 3^e^ et le 15^e^ percentile).

**Chronologie:** le développement psychomoteur a été décrit comme normal jusqu'à l'âge de 6 mois, suivi d'une régression progressive. Cette détérioration développementale a motivé plusieurs hospitalisations antérieures, sans qu'aucune orientation diagnostique précise n'ait été établie.

**Figure 1:**
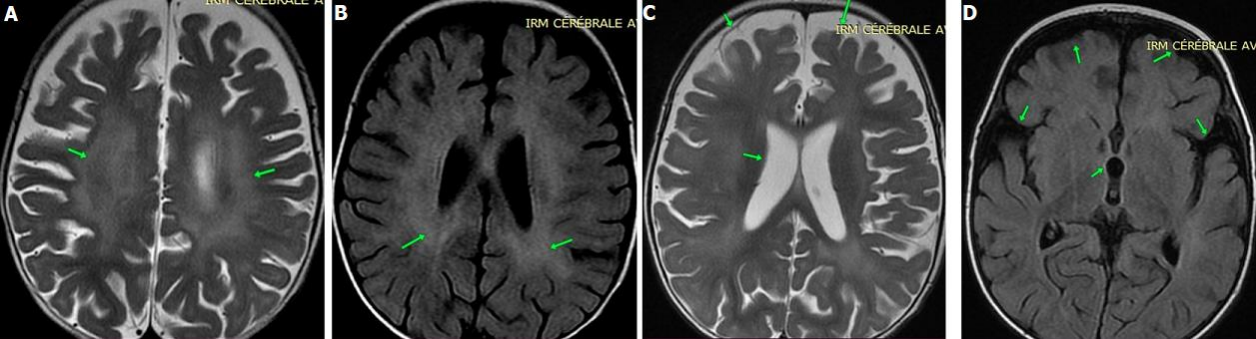
IRM cérébrale objective: coupe axiale séquence T2 (A), coupe axiale séquence flair (B) montrant une leuco-encéphalopathie hypersignal non expansif de la substance blanche; coupe axiale séquence T2 (C) et une coupe axiale séquence flair (D) montrant une atrophie cortico sous-corticale

**Démarche diagnostique:** face à ce tableau clinique non spécifique, une investigation exhaustive a été entreprise. Les examens neurophysiologiques (EEG de veille et de sommeil, ENMG, PEA et PEV) se sont tous révélés normaux. L'IRM cérébrale a montré une légère atrophie cortico-sous-corticale avec une leucoencéphalopathie hypersignal non expansive de la substance blanche sans calcifications visibles (Figure 1). Le bilan biologique standard et l'analyse du LCR étaient normaux, sans élévation notable de la cellularité ou de la protéinorachie. Le dosage de l'interféron alpha, biomarqueur caractéristique du SAG, n'a pas pu être réalisé pour des raisons techniques. L'exploration métabolique approfondie (ammoniémie, CPK, bilans hépatique et thyroïdien, recherche de toxiques et de métaux lourds) n'a pas révélé d'anomalies. Les analyses enzymatiques spécifiques pour les principales maladies de surcharge (sphingomyélinase, hexosaminidase A et B, arylsulfatase) se sont avérées normales à deux reprises. Les sérologies virales ont montré une positivité pour le cytomégalovirus compatible avec une infection ancienne non active.

L'association d'une régression psychomotrice, d'anomalies neuroradiologiques subtiles et l'exclusion méthodique des étiologies alternatives a conduit à évoquer le diagnostic de SAG. Une analyse génétique moléculaire ciblée a confirmé cette hypothèse en identifiant une mutation homozygote du gène RNASEH2B.

**Intervention thérapeutique:** en l'absence de traitement curatif, la prise en charge a été orientée vers une approche symptomatique multidisciplinaire visant à optimiser la qualité de vie et à prévenir les complications secondaires. Un programme de rééducation fonctionnelle intensive a été instauré. L'alimentation a été adaptée avec des textures modifiées pour compenser les troubles de déglutition. Le calendrier vaccinal a été complété, incluant la vaccination antigrippale pour minimiser le risque d'infections respiratoires.

**Suivi et résultats:** au terme d'une année de suivi régulier, l'enfant n'a pas développé de complications graves (infections sévères, crises convulsives, détresse respiratoire). Son développement reste néanmoins marqué par un retard psychomoteur persistant, sans progression significative des acquisitions motrices.

**Perspective du patient:** les parents de la patiente ont exprimé leur soulagement suite à l'établissement d'un diagnostic précis, après de longs mois d'incertitude et d'inquiétude. Ils ont souligné l'importance de la prise en charge multidisciplinaire et se disent satisfaits de l'accompagnement proposé, même en l'absence de traitement curatif.

**Consentement éclairé:** le consentement libre et éclairé des parents du nourrisson a été obtenu en vue de la préparation et de la publication du présent manuscrit.

## Discussion

Le cas présenté illustre parfaitement les défis diagnostiques posés par le syndrome d'Aicardi-Goutières, particulièrement lorsque la présentation clinique et radiologique s'écarte du phénotype classique [2]. L'absence de microcéphalie et de calcifications intracrâniennes chez notre patiente représente une déviation notable par rapport aux critères diagnostiques historiques du SAG [1,2]. La régression psychomotrice survenant après une période de développement apparemment normal constitue cependant un élément clinique caractéristique de la forme tardive du SAG, typiquement observée entre 3 et 12 mois [4]. Cette présentation temporelle est significativement associée aux mutations du gène RNASEH2B, comme l'ont documenté [3,5,6]. L'atteinte de ce gène, représentant environ 35-40% des cas, est généralement corrélée à un phénotype moins sévère, avec préservation relative du périmètre crânien et évolution neurologique plus favorable que les autres variantes génétiques [3,6]. Les troubles de déglutition et le stridor laryngé observés, bien que rarement décrits dans la littérature concernant le SAG, peuvent s'expliquer par une dysfonction bulbaire progressive dans le cadre de l'atteinte neurologique évolutive Ces manifestations méritent une attention particulière car elles constituent des facteurs de risque significatifs pour les complications nutritionnelles et respiratoires. L'absence d'engelures, considérées comme une manifestation extraneurologique caractéristique du SAG (particulièrement dans les mutations SAMHD1 et TREX1), est cohérente avec l'implication du gène RNASEH2B, dont les manifestations cutanées sont nettement moins fréquentes [5].

Notre démarche diagnostique, centrée sur l'exclusion méthodique des étiologies alternatives, est conforme aux recommandations actuelles qui positionnent le SAG comme un diagnostic d'exclusion dans les encéphalopathies précoces inexpliquées [3,8]. La confirmation par analyse génétique moléculaire a été déterminante, soulignant l'importance croissante du séquençage génétique dans l'identification des pathologies neurologiques rares aux présentations atypiques [3,8]. Le pronostic du SAG varie considérablement selon le génotype impliqué. Les mutations RNASEH2B sont généralement associées à une évolution clinique moins sévère comparativement aux autres variantes génétiques, notamment TREX1 ou RNASEH2A [3]. Cette observation pronostique semble se confirmer chez notre patiente qui, après un an de suivi, n'a pas développé de complications majeures.

Les avancées thérapeutiques récentes se concentrent sur l'inhibition de la voie de l'interféron de type I, notamment par l'utilisation d'inhibiteurs Janus kinase (JAK). Des essais cliniques sont actuellement en cours, offrant des perspectives prometteuses pour le traitement de cette pathologie jusqu'alors considérée comme exclusivement symptomatique [9].

## Conclusion

Ce cas atypique de syndrome d'Aicardi-Goutières souligne l'importance fondamentale d'intégrer cette entité dans le diagnostic différentiel des encéphalopathies pédiatriques précoces, même en l'absence des marqueurs cliniques et radiologiques classiques. L'hétérogénéité phénotypique du SAG, étroitement liée au génotype sous-jacent, justifie une approche diagnostique approfondie incorporant systématiquement l'analyse génétique moléculaire face à toute régression psychomotrice inexpliquée. Le diagnostic précoce, bien qu'il n'altère pas fondamentalement l'évolution naturelle de la maladie dans l'état actuel des connaissances thérapeutiques, permet néanmoins une prise en charge adaptée et un conseil génétique approprié. Les perspectives thérapeutiques émergentes ciblant spécifiquement la cascade inflammatoire médiée par l'interféron offrent désormais un espoir tangible pour les patients atteints de cette maladie rare.
